# Comparing the face inversion effect in crows and humans

**DOI:** 10.1007/s00359-017-1211-7

**Published:** 2017-09-13

**Authors:** Katharina F. Brecht, Lysann Wagener, Ljerka Ostojić, Nicola S. Clayton, Andreas Nieder

**Affiliations:** 1 Department of Psychology, University of Cambridge, Downing Street, Cambridge, CB2 3EB UK; 2 Animal Physiology, Institute of Neurobiology, University of Tübingen, Auf der Morgenstelle 28, 72076 Tübingen, Germany

**Keywords:** Face inversion, Corvid, Categorization, Delayed matching-to-sample, Social cognition

## Abstract

Humans show impaired recognition of faces that are presented upside down, a phenomenon termed face inversion effect, which is thought to reflect the special relevance of faces for humans. Here, we investigated whether a phylogenetically distantly related avian species, the carrion crow, with similar socio-cognitive abilities to human and non-human primates, exhibits a face inversion effect. In a delayed matching-to-sample task, two crows had to differentiate profiles of crow faces as well as matched controls, presented both upright and inverted. Because crows can discriminate humans based on their faces, we also assessed the face inversion effect using human faces. Both crows performed better with crow faces than with human faces and performed worse when responding to inverted pictures in general compared to upright pictures. However, neither of the crows showed a face inversion effect. For comparative reasons, the tests were repeated with human subjects. As expected, humans showed a face-specific inversion effect. Therefore, we did not find any evidence that crows—like humans—process faces as a special visual stimulus. Instead, individual recognition in crows may be based on cues other than a conspecific’s facial profile, such as their body, or on processing of local features rather than holistic processing.

## Introduction

Compared to stimuli of other categories, in humans, recognition and memory of faces is disproportionately impaired when faces are presented upside down even though both upright and inverted stimuli carry the same physical information (Yin 1969; Rhodes et al. 1993; Rossion 2009). While inversion reduces recognition of non-face stimuli by only 10%, recognition of faces is reduced by about 25% (Carey and Diamond 1977; Diamond and Carey 1986). This ‘face inversion effect' has been interpreted as an indicator for specialised or proficient processing of faces compared to other stimuli (Liu and Chaudhuri 2003), possibly reflecting a different mechanism (Farah et al. 1995).

Inversion of a stimulus impedes the configural processing of this stimulus (Towler and Eimer 2016), i.e., the encoding of spatial relations between different features, such as the distance between the eyes. Our ability to recognise faces is thought to rely on such configural (or holistic) processing (Bartlett and Searcy 1993; Rhodes et al. 1993; Collishaw and Hole 2000; Maurer et al. 2002). This configural processing appears early in life (Turati et al. 2004; Simion and Giorgio 2015) and seems to mature with time (de Heering et al. 2007; Cassia et al. 2009). Hence, some researchers argue that this domain-specific processing of faces is innate (Farah et al. 1995). However, others suggest it is also the result of our experience and thus reflects expertise for processing faces (Diamond and Carey 1986; Gauthier and Tarr 1997). This expertise is achieved by exploiting, where possible, a configural assembly of an object’s features, and can thus be achieved with any type of stimulus where individual stimuli share many similar features (Gauthier and Tarr 1997; Gauthier et al. 1998). Diamond and Carey (1986), for example, report that ‘dog experts’ show an inversion effect for dog pictures. Furthermore, certain non-face stimuli are sensitive to inversion, for example words or body postures (Reed et al. 2003). Thus, the face inversion effect is not necessarily confined to conspecific’s faces. Rather than reflecting a domain-specific process of face perception, the effect could be a result of expertise. Taken together, it has been argued that a specialised processing of faces might be due to an innate predisposition that matures with exposure (Simion and Giorgio 2015).

Arguably, humans process faces in a specialised manner because faces represent highly relevant cues offering a range of information about, for example, identity, age, sex, or emotional states of social partners (Todorov et al. 2008; Leopold and Rhodes 2010). However, humans are not the only animals that need to differentiate between individuals (Rosa Salva et al. 2015): the face inversion effect as an indicator for specialised face processing has also been investigated in non-human animals. Chimpanzees seem to exhibit a face inversion effect (Parr et al. 1998; Parr 2011a; Dahl et al. 2013), whereas research with rhesus monkeys reports more mixed results (Parr et al. 1999; Parr 2011b). This inconsistency has been attributed to the use of unsuitable methods (Dahl et al. 2013). Aside from primates, so far only a handful of other non-human species have been investigated. Socially living sheep, for example, are able to differentiate between faces of their conspecifics (Tate et al. 2006) and also show a face inversion effect (Kendrick et al. 1996), whereas pigeons do not (Phelps and Roberts 1994).

In the present study, the face inversion effect was investigated in crows. There are two reasons why corvids are an interesting model for studying the face inversion effect. First, corvids, similarly to humans and great apes, show a range of socio-cognitive abilities (e.g., Ostojić et al. 2013; Clayton and Emery 2015; Legg et al. 2015) that require them to differentiate between individuals in diverse contexts. For example, they might need to distinguish between different observers when protecting their caches from them—indeed, scrub-jays and ravens have been found to keep track of which individuals do and do not know about their caches and thus do or do not pose a threat to their caches (Dally et al. 2006; Bugnyar 2011). Furthermore, ravens are known to be aware of relationships between members of their social group (Massen et al. 2014) and adjust their willingness to cooperate with a partner based on identity (Massen et al. 2015). Thus, corvids seem to attend to the identity of their social partners both in cooperative and in competitive situations. Second, previous work suggests that corvids can recognise individuals (Kondo et al. 2012) and are also able to recognise conspecifics using visual cues alone: Rooks can differentiate between their partner and other conspecifics shown on video (Bird and Emery 2008), and carrion crows can be trained to differentiate between full-body pictures of conspecifics (Braun 2013). Hence, the ability to recognise conspecifics and the relevance of the identity of different conspecifics suggests that for corvids, conspecifics represent a relevant stimulus. Consequently, we aimed to assess a potential face inversion effect for conspecific faces as an indicator of specialised processing of faces.

Given the repeated exposure of captive crows to human faces, crows might have developed an expertise for human faces, similarly to humans who developed an expertise for dogs (Diamond and Carey 1986). Hence, our second aim was to investigate whether another stimulus of everyday relevance for captured crows could elicit a face inversion effect: the human face. Previous research supports this prediction because both hand-raised (von Bayern and Emery 2009) and wild corvids (Marzluff et al. 2010; Clucas et al. 2013) have been found to attend to human faces. Furthermore, American crows recognise humans based on their face more than 2 years after the initial presentation (Marzluff et al. 2010) and can differentiate between male and female human faces from coloured pictures (Bogale et al. 2011). Thus, it is likely that crows can use facial cues to differentiate between humans.

To test the hypothesis that birds of the crow family show performance disruption when recognising inverted compared to upright faces, we administered a delayed matching-to-sample task to carrion crows in Experiment 1. Specifically, we compared performance when crows had to recognise: (1) crow faces and non-face control stimuli (side view of a fish), both inverted and upright and (2) human faces and non-face control stimuli (interior of a house). Non-face controls were chosen based on their similarity to the human/crow face stimuli. If faces are ‘special’ for crows, they should have an impaired performance for inverted images compared to upright images. This impaired performance should further be more pronounced when responding to faces compared to when responding to non-face stimuli. In Experiment 2, we compared the crows’ performance to that of human participants using the same stimuli and setup.

## Materials and methods

A possible face inversion effect was investigated in a delayed matching-to-sample task. Two crows and 20 human participants were tested. In the following, we outline the procedures and setup used for both crows (Experiment 1) and humans (Experiment 2).

### Investigating a face inversion effect in carrion crows (Experiment 1)

#### Subjects and housing

Two male carrion crows, aged 3 years (Walt) and 2 years (Hugo), participated in the experiment. The crows were housed in large indoor aviaries (360 × 240 cm × 300 cm) side by side in groups of four at the Animal Physiology lab, University of Tübingen, Germany. The crows had been taken from the institute’s breeding stock (Hoffmann et al. 2011). The birds were kept on a controlled feeding protocol for the duration of the experiment and earned food during and, if necessary, after the daily tests. Body weight was measured daily. Outside of testing, the birds’ diet consisted of chick meat and mashed birdseeds. Water was provided ad libitum in the aviary and during testing. Training and data collection lasted from July to October 2016. All experimental procedures were approved by the local ethical committee and authorised by the national authorities (Regierungspräsidium Tübingen).

#### General procedure

**Fig. 1:**
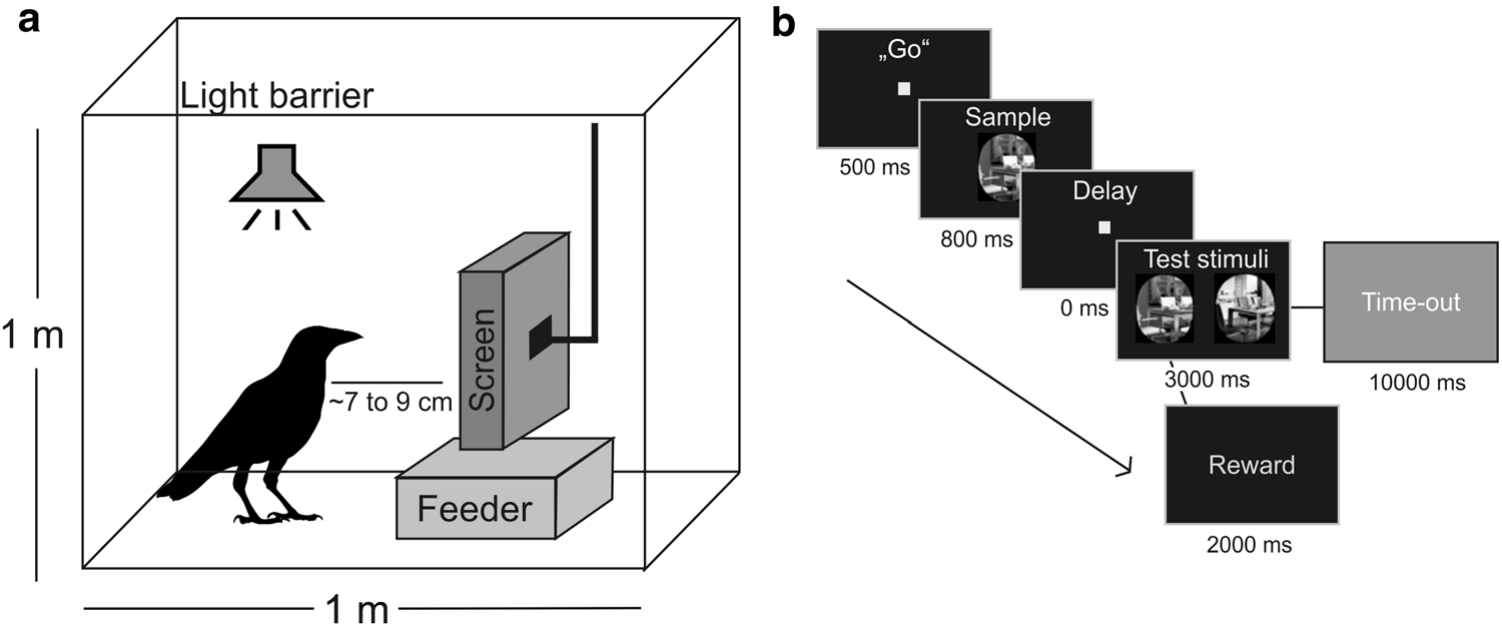
**a** Set-up for Experiment 1. Crows sat in an operant conditioning chamber measuring 100 × 76 × 100 cm. During testing, the doors of the chamber were kept closed to minimise disruption and to avoid reflections on the screen. **b** Delayed matching-to-sample task used in Experiment 1 and 2. Presentation times varied depending on training progress

The birds were trained and tested on the matching-to-sample task in a darkened operant conditioning chamber (Fig. 1a). The CORTEX program (National Institute of Mental Health, MD, USA) was used for stimulus presentation and measuring the birds’ performance as error rates. Visual stimuli were displayed on a touch screen monitor (ART development PS-150, 15’’, 60-Hz refresh rate), allowing the birds to respond by pecking at stimuli shown on the screen. Leather jesses secured birds loosely to their perch.

Rewards (Beo Special pearls or mealworm beetle larva) for 75% of correct trials were delivered with an automated feeder below the screen. Additionally, birds received auditory feedback with specific tones for correct and incorrect trials. Birds could initiate a trial by placing their head in an infra-red light barrier: in combination with a reflector foil attached to the birds’ head the light barrier was activated when the birds were positioned in front of the screen and facing it. Trials were aborted and not counted when the crow left the light barrier during sample presentation. The retainer of the reflector of the light barrier was implanted under general anaesthesia onto the birds’ skull for experiments conducted prior to the present study. For a description of surgical procedures, see, e.g., Veit and Nieder (2013). A Go-stimulus (a small white square) was presented on the screen to indicate a new trial (Fig. 1b). A short click indicated the activation of the light barrier and the Go-stimulus disappeared (pre-sample phase). Next, the birds saw a sample stimulus at the centre of the screen (i.e., one of the images described below). After a short delay, two test stimuli, the match and the non-match stimuli, were shown left and right of the centre. The birds had to respond within 3000 ms by pecking one of the stimuli. During training, the delay between sample and test stimuli as well as time-out after incorrect responses were adjusted depending on performance.

In case of an incorrect response, the particular trial was presented in a delayed and pseudo-randomized way until all stimuli combinations were shown once. Only during training, but not during data collection, the retry occasionally took place immediately after an incorrect trial. This was done when birds started to develop a side bias or when performance dropped to chance level once a new stimulus type was introduced.

Birds received between 300 and 480 correct trials a day during training.

#### Material

The pictures used had been downloaded from google images and flickr.com. Pictures of human faces were selected with permission from the face database provided by the Max Planck Institute of Biological Cybernetics in Tübingen, Germany (Troje and Bülthoff 1996). Pictures of all stimuli were achromatic and brightness was equalised. Pictures were between 45 × 43 and 77 × 47 pixels in size. When performing the tasks, the distance between the birds’ eyes and the screen was around 7 cm (Walt) and 9 cm (Hugo), creating an angular diameter of 17.1 and 16.3, respectively.

**Fig. 2:**
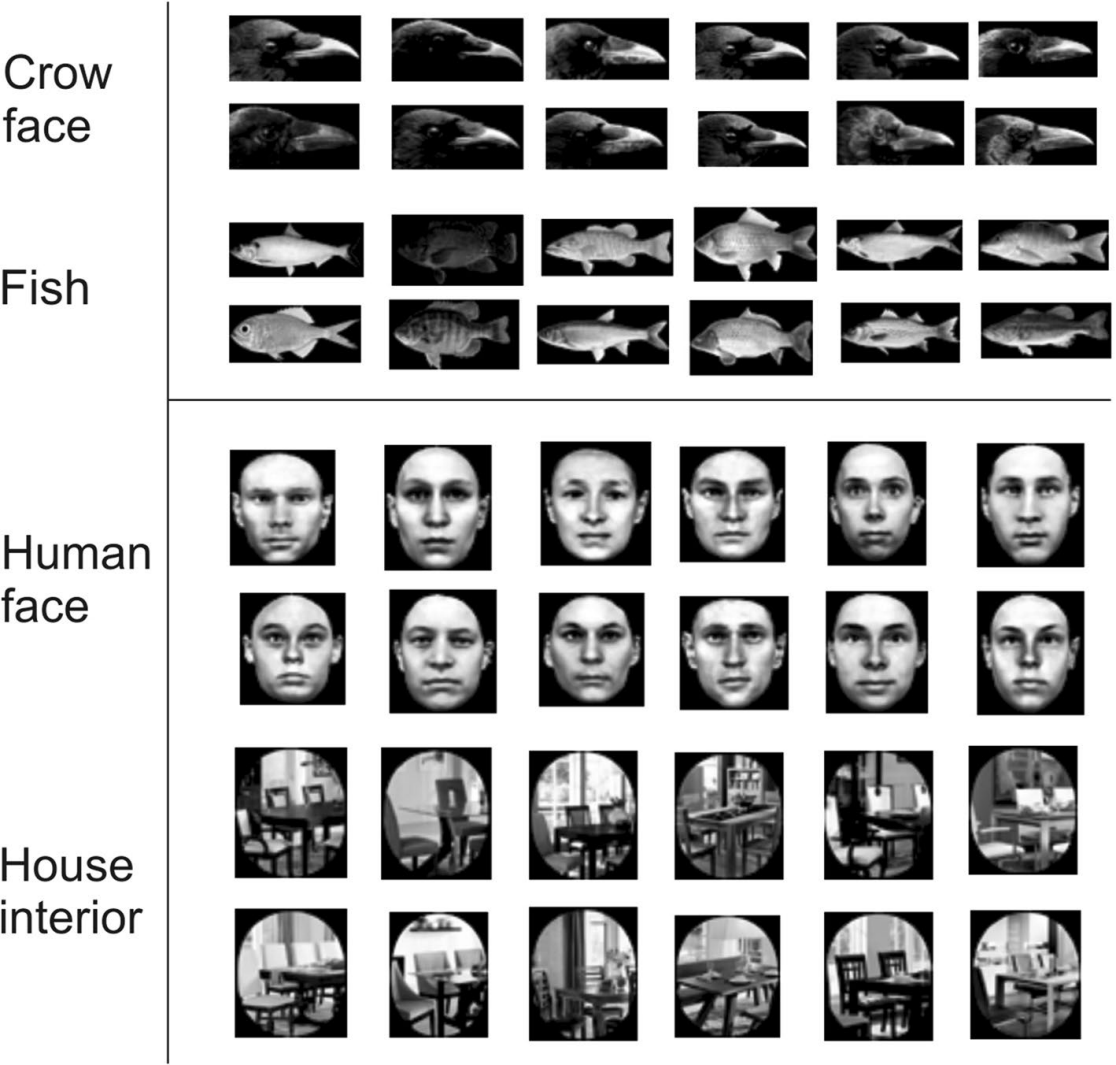
Stimuli used for testing. Crows were tested on four categories of stimuli: crow faces and corresponding controls (i.e., fish), and human faces and corresponding controls (i.e., house interior)

For data collection, four different categories of stimuli were used (Fig. 2): profiles of crow faces, human faces, house interiors, and fish. The pictures of the crow profile were from different individuals and of the fish from different species of fish. The crows were not familiar with the crows depicted. There is some indication that for jungle crows the shape of the beak might be used to discriminate between individuals (Kondo and Izawa 2014). Consequently, due to the loss of information about beak size and shape when viewed frontally, the profile might be relevant when recognising conspecifics. Indeed, previous research showed that birds recognise faces in full or ¾ profiles (Trillmich 1976; Brown and Dooling 1992). Moreover, crows rarely see a frontal view of conspecific faces due to their visual scanning behaviour (Fernández-Juricic et al. 2010). Hence, in the present study profiles of carrion crows’ heads were used rather than their faces. Note that using the profile was also a practical decision: it was not feasible to acquire a range of portraits of crows facing straight forward. One reason for this might be that corvids exhibit a lot of head movements to scan their environment (Fernández-Juricic et al. 2010) and thus rarely look straight into a camera.

Pictures of fish served as non-face controls for the crow faces and pictures of house interiors as non-face controls for the human faces. Fish were used as non-face controls for two reasons: first, pictures of different fish species were readily available in the same orientation (profile). Second, regardless of the hypothesis about the origin of the face inversion effect is adopted, fish should not be configurally processed by carrion crows: if configural processing of faces is innate, only conspecifics should be relevant for crows, and if it is due to specialised expertise, fish should only be configurally processed by crows who have repeated exposure to fish and have a reason to differentiate between different species of fish. All pictures were presented both upright and inverted.

#### Behavioural protocol

Both crows had previously participated in other experiments using the same set-up and were thus habituated to the set-up and general procedure.

#### Matching-to-sample task

Several training steps were applied. First, the crows had to match colours (blue and red) and chromatic ‘abstract’ pictures taken from Veit and Nieder (2013) until they reached criterion (defined as accuracy >70%). In Step 2, birds had to match achromatic abstract patterns. In Step 3, birds had to match achromatic pictures of the same category (e.g., footballs). In Step 4, birds had to recognise two pictures of four different categories (mugs, tires, flowers, and keys).

#### Data collection

During data collection, six pairs of stimuli per class were used. Each correct test stimulus appeared once on the right and once on the left side of the screen, and each stimulus was twice the match stimulus and twice the non-match stimulus. Trial order was blocked, such that pictures of one category were blocked together. The order of blocks and trials within each block was randomised.

The crows were presented with a minimum of 192 correct trials during a session (4 different pairings per stimuli × 6 stimuli pairs × 2 orientations × 4 stimuli categories). Therefore, crows saw each picture at least 4 times during one session. During data collection, crows received between 384 and 576 trials each day (2–4 sessions).

#### Analysis

Data were extracted from CORTEX (National Institute of Mental Health) using MATLAB R2016a. For data analyses, a difference index was calculated for the percentage of correct responses on upright minus the percentage of correct responses on inverted trials (DI = Upright − Inverted). A face inversion effect would predict a larger impairment of the crows’ performance when responding to face compared to non-face stimuli. Hence, the DI should be larger in face than non-face categories.

Data were analysed for each crow separately. First, the DI (as performance for upright stimuli minus the performance for inverted stimuli) when responding to crow faces was compared to the DI when responding to non-face controls (fish pictures), DI_crow face_ > DI_fish_. Second, the DI when responding to human faces was compared to the DI when responding to non-face controls (house interior pictures), DI_human face_ > DI_house interior_.

Whether overall performance differed from chance or not was analysed using a binomial test in RStudio Version 1.0.136 (R Core Team 2016). To assess the face inversion effect, the proportion of correct responses to all pictures of one category was calculated as one score for each category during each session. This was done for both upright and inverted stimuli separately. The comparisons between DI_crow face_ > DI_fish_ as well as between DI_human face_ > DI_house interior_ were analysed with paired Wilcoxon rank tests in RStudio. Comparisons based upon clear predictions were calculated using directional (one-sided) tests (Ruxton and Neuhäuser 2010).

All analyses were based upon clear predictions and as such all comparisons were calculated using directional (one-sided) tests (Ruxton and Neuhäuser 2010).

### Investigating the face inversion effect in human participants (Experiment 2)

#### Participants

Twenty participants were recruited and tested at the Institute of Biology at the University of Tübingen, Germany, aged 21–35 (*M* = 26.7), of which 13 were females. The experiment was performed with the approval of the Ethical Committee of the Faculty of Science, University of Tübingen, performed in accordance with the ethical standards laid down in the 1964 Declaration of Helsinki. Data were collected from January to February 2017.

#### Set-up and material

The same test stimuli as in Experiment 1 were used. The set-up was the same as in Experiment 1 except that the touch screen was moved to face the participants sitting in front of the box. The room was darkened. Piloting the original task on KFB and LW showed that humans were likely to perform at ceiling if the same timings as in the crow task were used. Thus, the presentation time of the sample was reduced to 500 ms and the delay between sample and test stimuli was increased to 500 ms. Furthermore, the available response time until a trial was aborted was reduced to 710 ms.

#### Procedure

Participants were instructed verbally. They were asked to complete 192 correct trials each. Similarly to the crows, humans received a retry for incorrect trials. The experiment took 20 min in total.

#### Analysis

Data were extracted from CORTEX (National Institute of Mental Health) using MATLAB R2016a and were analysed in RStudio Version 1.0.136 (R Core Team 2016). For data analyses a difference index was calculated for percentage of correct responses in upright minus inverted trials (DI = upright − inverted).

Due to non-normality, data were analysed using Wilcoxon signed rank tests. Because the analysis was based on clear predictions, directional tests were used. Cohen’s *d*s were corrected for dependence according to Morris and DeShon (2002). First, the DI (as performance for upright stimuli minus the performance for inverted stimuli) when responding to crow faces was compared to the DI when responding to non-face controls (fish pictures), DI_crow face_ > DI_fish_. Second, the DI when responding to human faces was compared to the DI when responding to non-face controls (house interior pictures), DI_human face_ > DI_house interior_. Additionally, we compared the performance on trials with crow faces with the performance on human faces, regardless of orientation.

## Results

### Assessing a possible face inversion effect in carrion crows

**Fig. 3:**
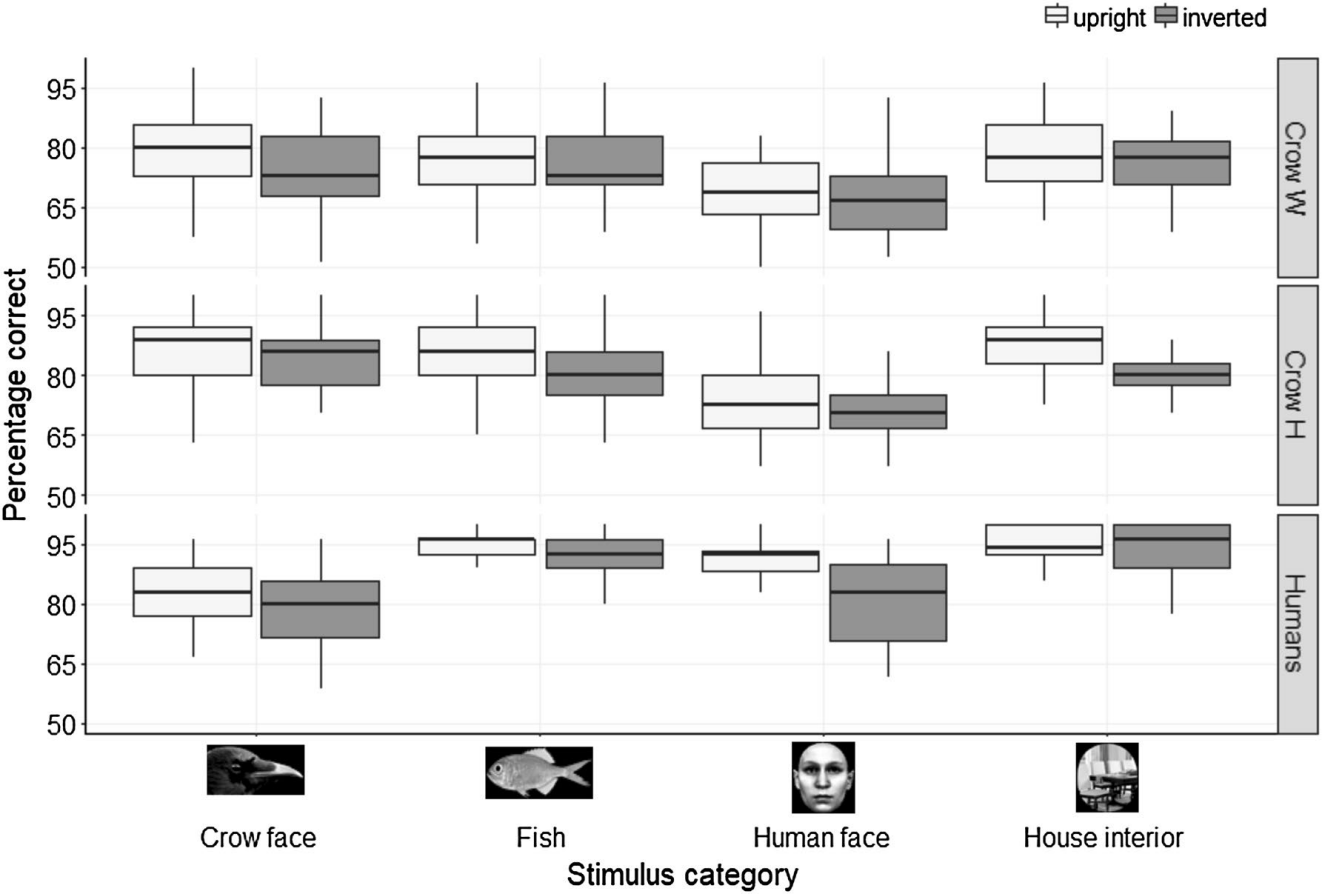
*Box*-and-*whiskers* plot showing the performance for all stimulus categories when responding to upright stimuli (*light grey*) and inverted stimuli (*dark grey*) for crow Hugo (*n* = 37 sessions), crow Walt (*n* = 39 sessions) and the human participants (*n* = 20). The *boxes* signify the *upper* and *lower* quartiles and the *thick black horizontal lines* the median. The whiskers extend from the box to values no further than ±1.5 * IQR from the *box*

**Fig. 4:**
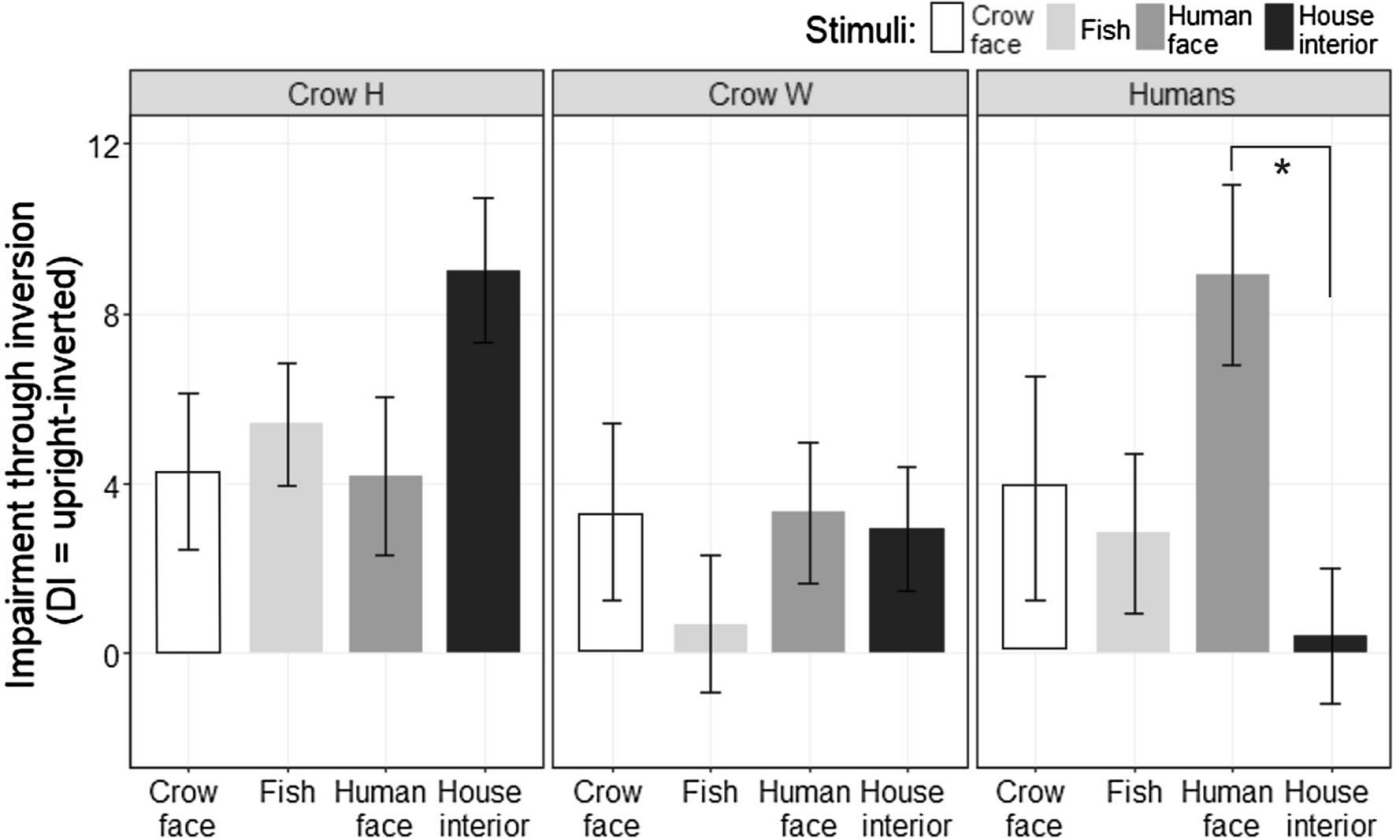
Mean DI in performance ± SEM. Performance scores when responding to inverted pictures were subtracted from the performance scores when responding to upright pictures to determine the impairment due to inversion for the different stimulus categories, comparing crow Hugo, crow Walt and human participants. The *asterisk* indicates a significant difference (*p = .001, Wilcoxon-signed rank test)

We first assessed the presence of a putative face inversion effect in crows (Experiment 1). Figure 3 gives the performance scores of the two crows for all categories in upright and inverted trials. Both crows performed the task better than chance (50%) for all stimulus categories (Binomial tests, all *p*’s < .001). Crow Hugo scored on average *M* = 86.9% (SD = 9.1%) on trials with upright crow faces, and *M* = 82.6% (SD = 9.4%) on trials with inverted crow faces, DI_crow face_ = 4.3%. He scored on average *M* = 86.0% (SD = 7.6%) on trials with upright non-face controls (fish), and *M* = 80.6% (SD = 7.6%) on trials with inverted non-face controls, DI_fish_ = 5.4%. Hence, as can be seen in Fig. 4, Hugo did not show a face-specific inversion effect for crow faces, *U* = 365.5, *p*_one-sided_ = .698. With the human faces, he scored on average *M* = 73.8% (SD = 8.9%) on trials with upright human faces, and *M* = 69.6% (SD = 8.0%) on trials with inverted human faces, DI_human faces_ = 4.2%. He scored on average *M* = 88.1% (SD = 7.8%) on trials with upright non-face controls (house interior), and *M* = 79.1% (SD = 7.8%), on trials with inverted non-face controls, DI_house interior_ = 9.0%. Thus, Hugo also did not show a face-specific inversion effect for human faces, *U* = 469, *p*_one-sided_ = .962.

Crow Walt scored on average *M* = 77.4% (SD = 10.0%) on trials with upright crow faces, and *M* = 74.1% (SD = 10.5%) on trials with inverted crow faces, DI_crow face_ = 3.3%. He scored on average *M* = 76.0% (SD = 9.9%) on trials with upright non-face controls (fish), and M = 75.3% (SD = 8.1%) on trials with inverted non-face controls, DI_fish_ = 0.7%. This difference in DI did not reach significance, *U* = 267, *p*_one-sided_ = .070, see Fig. 3. With the human faces, he scored on average *M* = 69.8% (SD = 8.8%) on trials with upright human faces, and *M* = 66.4% (SD = 9.6%) on trials with inverted human faces, DI_human faces_ = 3.3%. He scored on average *M* = 78.8% (SD = 9.5%) on trials with upright non-face controls (house interior), and *M* = 75.8% (SD = 9.5%), on trials with inverted non-face controls, DI_house interior_ = 2.93%. Thus, Walt also did not show a face-specific inversion effect for human faces, *U* = 226, *p*_one-sided_ = .339.

Interestingly, as can be seen in Fig. 3, as well as in Table 1, both crows performed better when crow faces were presented compared to when human faces were presented, regardless of orientation (Hugo: *U* = 2408.5, *p*_two-sided_ < .001; Walt: *U* = 2101, *p*_two-sided_ < .001). Furthermore, both crows generally performed better when responding to upright than to inverted stimuli, regardless of category (Hugo: *U* = 7785, *p*_two-sided_ < .001; Walt: *U* = 6667, *p*_two-sided_ = .004).

**Table 1:** Overview of performance (percentage of correct choice) for all stimulus categories for both birds and human participants, averaged across all sessions

	Mean (SD) performance in %		
	Crow Hugo	Crow Walt	Human participants
Fish	83.3 (8.0)	75.6 (9.0)	92.3 (6.1)
House interior	83.6 (8.8)	77.3 (9.0)	94.3 (5.8)
Crow face	84.7 (9.5)	75.8 (10.3)	80.6 (10.1)
Human face	71.7 (8.7)	68.1 (9.3)	85.8 (10.0)
Upright	83.7 (10.1)	75.5 (10.1)	90.3 (8.0)
Fish	86.0 (7.7)	76.0 (9.9)	93.7 (5.4)
House interior	88.1 (7.8)	78.7 (9.5)	94.5 (4.5)
Crow face	86.9 (9.1)	77.4 (10.0)	82.5 (8.7)
Human face	73.8 (8.9)	69.8 (8.8)	90.2 (6.7)
Inverted	78.0 (9.5)	72.9 (9.8)	86.2 (11.0)
Fish	80.6 (7.6)	75.3 (8.1)	90.9 (6.5)
House interior	79.1 (7.8)	75.8 (8.3)	94.1 (7.0)
Crow face	82.6 (9.4)	74.1 (10.5)	78.7 (11.1)
Human face	69.9 (7.9)	67.0 (9.5)	81.2 (10.9)
Overall	80.8 (10.2)	74.2 (10.0)	88.2 (9.8)

### Validation of the methodology with human participants

We validated the face inversion effect in humans using the same methodology (Experiment 2). The percentage of correct responses was on average *M* = 82.5% (SD = 11.1%) on trials with upright crow faces, and *M* = 78.7% (SD = 8.72%) on trials with inverted crow faces, DI_crow face_ = 3.88%. On trials with upright non-face controls (fish), the percentage of correct responses was on average *M* = 93.7% (SD = 5.4%), and *M* = 90.9% (SD = 6.5%) on trials with inverted non-face controls, DI_fish_ = 2.83%. This difference in DI did not reach significance, *U* = 114.5, *p* = .222 (Fig. 4). For the human faces, the average of percentage of correct responses was *M* = 90.2% (SD = 6.7%) on trials with upright stimuli, and *M* = 81.3% (SD = 10.9%) on trials with inverted stimuli, DI_human faces_ = 8.91. On trials with upright non-face controls (house interiors), the average of percentage of correct responses was *M* = 94.5% (SD = 4.5%), and *M* = 94.1% (SD = 7.0%), on trials with inverted non-face controls, DI_house interior_ = 0.41%. The DI_human face_ was significantly larger than DI_house interior_, *U* = 170.5, *p* = .001. Hence, as can be seen in Fig. 4, humans showed a face inversion effect for human faces.

## Discussion

In this study, we present results suggesting that carrion crows do not exhibit a face inversion effect. The face inversion effect refers to a pronounced impairment in the ability to recognise and remember faces compared to other stimuli once the pictures are turned upside-down (Yin 1969; Diamond and Carey 1986). As such, the face inversion effect has been suggested to reflect a special processing of faces.

### The lack of a face inversion effect in carrion crows

In Experiment 1, we investigated whether carrion crows also show the face inversion effect or not, both with crow faces and with human faces. The crows performed better with upright than inverted stimuli in general, and their accuracy for inverted stimuli never reached the accuracy shown for upright stimuli. Some impairment following inversion is also found in humans (e.g., Diamond and Carey 1986; Experiment 2), and was previously reported for animals, too (e.g., Wright and Roberts 1996). However, note that this result could in part be explained by the fact that prior to data collection, when we selected the control stimuli, we exposed the crows to the upright examples of the respective category to assess which stimulus categories they were able to discriminate. One possible explanation for their difficulties to achieve similar performance for the inverted stimuli could be that they developed a strategy to respond to these pictures, which was rendered suboptimal once the pictures were inverted.

Furthermore, while not being the main focus of this study, it should be noted that crows were better at recognising crow faces compared to recognising human faces. However, neither of the crows tested showed a more pronounced impairment of their performance when presented with inverted faces—either human faces or crow profiles—compared to inverted control stimuli. Hence, the two crows tested did not show evidence of a face inversion effect.

There are three reasons why this lack of a face inversion effect in crows may be surprising. First, corvids can and need to identify specific individuals (e.g., Dally et al. 2006; Bugnyar 2011; Massen et al. 2015) and can do so from static pictures (e.g., Bird and Emery 2008; Braun 2013). Second, corvids can also recognise specific human faces (Marzluff et al. 2010; Clucas et al. 2013). And last, corvids can learn to discriminate pictures in general (Veit and Nieder 2013; Veit et al. 2014), and pictures of conspecifics in particular, as shown in Experiment 1. In the following, the lack of a face inversion effect in our crows is discussed in relation to the stimuli used and the cues crows (might) use to differentiate individuals.

### Positive validation of the experimental procedures in human adults

In order to directly test whether the stimuli used could have been responsible for the lack of a face inversion effect in carrion crows, Experiment 2 validated the procedure and stimuli used in Experiment 1 by testing humans in the same set-up and with the same stimuli as the crows. Whether a face inversion effect is present in animals or not is still a matter of debate. It has been argued that the conflicting results reported regarding whether or not primates show a face inversion effect is due to differences in methods and stimuli used (Dahl et al. 2013). For example, some studies used natural pictures of full primate heads, sometimes with some scenery in the background (e.g., Parr et al. 1999; Phelps and Roberts 1994; Wright and Roberts 1996), while newer studies have used very controlled pictures, showing only a face without any surrounding that might allow viewers to determine head shape (Dahl et al. 2013). Thus, in Experiment 2, the paradigm and stimuli used in Experiment 1 were validated with a human sample. Here, humans showed a strong face inversion effect: their performance in recognising faces was impaired to a greater extent when pictures of human faces were inverted compared to pictures of non-face controls. This result is in line with a range of previous studies on the face inversion effect in humans (e.g., Yin 1969; Diamond and Carey 1986; Kanwisher et al. 1998; Freire et al. 2000; Turati et al. 2004). The result of Experiment 2 further suggests that, in principle, the stimuli used in our study are appropriate to induce a face inversion effect, as they do so in human participants. Consequently, the null result in Experiment 1 cannot be explained by a methodological problem and instead reflects a lack of a face inversion effect in the crows. The consistent results from the two crows suggest that this species does not show a face-specific inversion effect. However, given the small sample size in the current study, it remains a possibility that our results might not apply to crows in general.

### Implications regarding the cues used by crows for individual recognition

Given the positive validation of the procedures used in Experiment 2, there are two possible reasons for a lack of face inversion effect in crows: first, crows might use and process cues other than face profiles to recognise and discriminate between conspecifics. It is not yet known whether crows use facial cues to identify conspecifics. There are reports of certain bird species using facial cues to discriminate between conspecifics (e.g., Trillmich 1976; Brown and Dooling 1992; Nakamura et al. 2003), for example the diverse plumage of the face (Leopold and Rhodes 2010). It is, however, possible that crows in the wild use the whole body as a cue, rather than the face alone. Notably, research on conspecific discrimination in crows has so far mainly used whole bodies (Braun 2013). Thus, it would be of interest to see whether crows have a ‘body-inversion’ effect. Reed et al. (2003) found that humans display a body inversion effect in that their performance in recognising human bodies is impaired by inversion whereas recognition of houses is not.

Another cue that corvids might use for identity discrimination is ultraviolet differences in plumage. Ultraviolet light perception has been reported to be relevant for mate choice in a range of bird species (for a review see Rajchard 2009, but for opposing views see Stevens and Cuthill 2007). For example, Steller’s jays’ plumage UV reflection signals mate quality. Note, however, that extra-pair copulations play a relatively important role for Steller’s jays, compared to other corvid species (Overeem et al. 2014). It is thus unclear whether the importance of UV perception in Steller’s jays’ sexual behaviour is indicative of visual features that might be relevant for monogamous and largely unassisted breeding carrion crows. Still, it is worth noting that the failure to find a face inversion effect might be due to the lack of UV light of the crow face stimuli used in the current study.

Second, the face inversion effect might simply not be an indicator for specialised processing in crows, maybe because crows do not process faces in a configural manner. In this case, a different approach might be necessary to evaluate whether faces are processed differently to stimuli of other categories. With electrophysiological experiments it has been demonstrated that rhesus macaques possess neural circuits specifically dedicated to processing faces (Allison et al. 2000; Gross 2008; Freiwald and Tsao 2010). Yet, studies investigating the face inversion effect in monkeys produced mixed results (e.g., Phelps and Roberts 1994; Wright and Roberts 1996; Parr 2011b). Thus, it has been argued that in monkeys, specialised face processing might not manifest itself in configural processing, which is susceptible to inversion (Leopold and Rhodes 2010). Consequently, it would be of interest whether electrophysiological experiments could uncover face-specific responses in the crow brain, too. Face-selective cells have been previously found in a range of primate species, from humans (Kanwisher and Yovel 2006) to macaques (Gross 2008; Freiwald and Tsao 2010) and marmoset (Hung et al. 2015), but also in a non-primate mammal, the sheep (Tate et al. 2006). Furthermore, faces seem to be special for another species of bird: newborn domestic chicks have been reported to show a predisposition to imprint on face-like stimuli (Johnson and Horn 1988; Rosa-Salva et al. 2010; Salva et al. 2011; Rosa Salva et al. 2012; Di Giorgio et al. 2016; Versace et al. 2017). It is thus possible that corvids, while not showing a face inversion effect, might have similar face-selective cells indicative of a specialised processing of faces.

### Specialised processing of human faces by crows?

Previous research suggests that crows can use the face of a human to differentiate between individuals (Marzluff et al. 2010; Bogale et al. 2011) and can be trained to discriminate between male and female faces based on pictures (Bogale et al. 2011); therefore, we assessed the face inversion effect in crows for human faces as well. However, this prior research alone does not imply that human faces constitute a ‘special’ cue for crows. This notion is tentatively supported by the results presented here, because the birds did not show an inversion effect when presented with human faces, suggesting that crows might use local features to differentiate them. Such feature recognition would not be impaired by inversion. There are of course a range of different features they could have used, such as for example the shape or size of the eyes. Future research is needed to assess whether they indeed used local features to solve the matching-to-sample task, and if so, which ones.

## Conclusion

In summary, our results suggest that crows do not exhibit a face inversion effect. We further show that crows can learn to discriminate between human as well as crow faces, and make fewer errors when responding to crow faces. Based on the rationale from human and other primate studies, these findings may be taken to mean that crows are no ‘experts’ for faces and thus do not process faces in a different way to other stimuli. Further research is needed to determine which cues crows use to differentiate between different conspecifics as well as humans, and whether there are other ways to assess a possible specialised processing of faces in crows.
